# A Rare Case of Perineurioma of the Posterior Interosseous Nerve: A Case Report

**DOI:** 10.7759/cureus.75856

**Published:** 2024-12-17

**Authors:** Kewithinwangbo Newme, Sumanjit Boro, Deb K Boruah, Yash Mehandiratta

**Affiliations:** 1 General Surgery, All India Institute of Medical Sciences, Guwahati, Guwahati, IND; 2 Plastic Surgery, All India Institute of Medical Sciences, Guwahati, Guwahati, IND; 3 Radiodiagnosis, All India Institute of Medical Sciences, Guwahati, Guwahati, IND; 4 Diagnostic and Interventional Radiology, All India Institute of Medical Sciences, Guwahati, Guwahati, IND

**Keywords:** intraneural perineurioma, perineurioma, perineurioma perineurioma, peripheral nerve sheath tumors, schwannoma

## Abstract

The perineurioma (PN) is a benign neoplasm with perineural origin. It can be of two types, i.e., intraneural PN and extraneural PN. It is slow-growing in nature and frequently causes hypoesthesia and progressive motor weakness other than swelling. The size of the swelling ranges from small to large. The entity is difficult to distinguish from Schwannoma and other peripheral nerve sheath tumors clinically, pathologically, and radiographically (MRI). An early diagnosis is needed as the PN arises from a nerve and can be salvable if preoperative planning is well executed. Here, in this case, report, the nerve involved was the posterior interosseous nerve (main motor nerve of the finger/wrist extensors), which is a rare phenomenon of occurrence; however, early intervention and the benign nature of the tumor can be handled with a good prognosis, and recurrence is usually rare but could not be ignored as documented.

## Introduction

Perineurioma (PN) is a rare slow-growing benign peripheral nerve sheath tumor that originates from perineurial cells [1]. It has two types, namely, extraneural and intraneural, and has distinctive features. The intraneural type usually involves the peripheral nerve boundaries, and the extraneural PN is usually the soft tissues and skin.

Da Gama Imaginario in 1964 first identified the intraneural PN and described it as benign and characterized by the complex ultrastructural similarity to schwannoma and neurofibroma. Moreover, histologically, it appears as perineurial cell proliferation, concentrically surrounding the peripheral nerve, typically appearing as pseudo-onion bulbs. Charcot-Marie tooth disease and chronic inflammatory demyelinating polyneuropathy have a similar trait of onion bulbs-like shaped histologically. An immunohistochemistry study can confirm the diagnosis of intraneural PN, especially positive for epithelial membrane antigen (EMA) and negative for S-100 [2-4].

PN involving the cutaneous and subcutaneous tissues may show a plexiform growth pattern, which may misinterpreted with other plexiform variants lesions, such as naevi, schwannoma, etc. Interestingly, similar plexiform variants are recognized as nerve sheath myxoma/neurothekeoma, Pacinian neurofibroma, and perineurial myxoma by various authors [5].

The PN involves both upper and lower limbs in equal proportion. It can also involve the trunk, head and neck, retroperitoneum, and paratesticular region [6]. The total number of PN cases recorded in the literature is around 172 cases to date. The nerves usually involved are sciatic, radial median, brachial plexus, peroneal, ulnar, tibial, posterior interosseous, femoral, cranial nerve, spinal roots, facial, sacral roots, sural, and other branches. The most commonly involved nerve is the sciatic sciatic. The documented total number of cases with posterior interosseous nerve involvement is five cases to date [7-9].

Usually, it presents as a mononeuropathy, which is insidious in onset with motor predominant and slow in progression and equally affects both genders and usually predilection for young adults. In the long course, it causes neurological deficits such as hypoesthesia, motor weakness, etc. The lesion is difficult to diagnose, and at the same time, the management is also challenging [7,10].

## Case presentation

The 38-year-old gentleman presented in OPD with right forearm swelling for the last five years, but he noticed an increase in the size of the swelling in the last six months. There was no tingling sensation or weakness on the involved side. However, he had a dull aching type of pain over the swelling for the last two months. On clinical examination, the swelling was noted on the right forearm dorsal aspect, 8 cm x 6 cm in size, and 5 cm distal to the right elbow joint. The swelling was non-mobile and non-tender, and there was no skin fixity. Multiple superficial skin lesions were noted as the patient had put herbal medicine over the swelling. There was no weakness or tingling sensation in the involved forearm.

**Figure 1:**
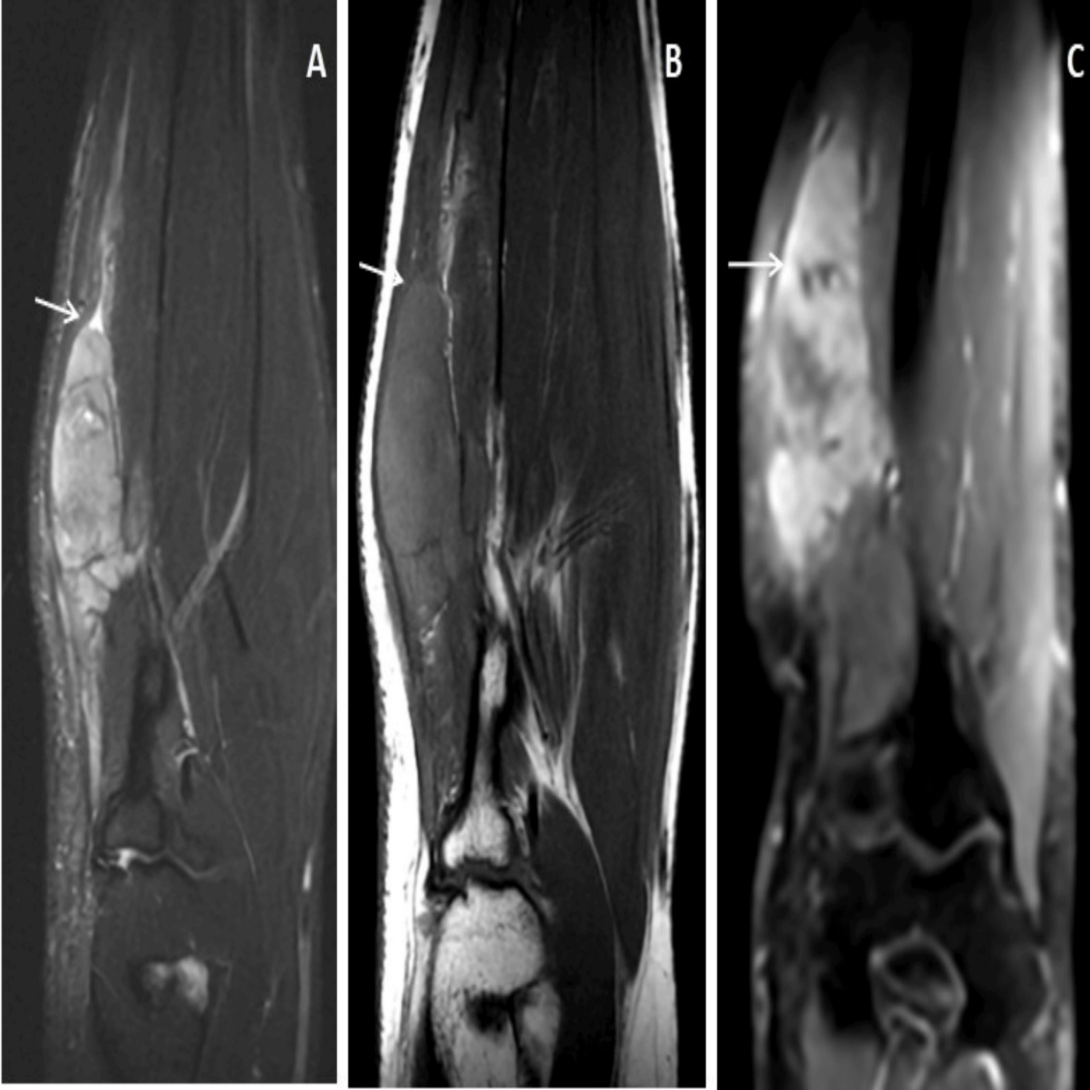
MRI images of the right forearm of the patient. The coronal proton density fat saturation (PDFS) and coronal T1-weighted (T1W) images (A and B) show a lobulated T1 isodense and PDFS hyperintense elongated appearing mass lesion seen in the radial aspect of the forearm infiltrating into the brachioradialis muscle. A positive fat sign is seen within the lesion (arrow in images A and B). On coronal post-contrast, the T1W image (C) shows the heterogeneously enhanching lesion with few non-enhancing cystic components.

**Figure 2:**
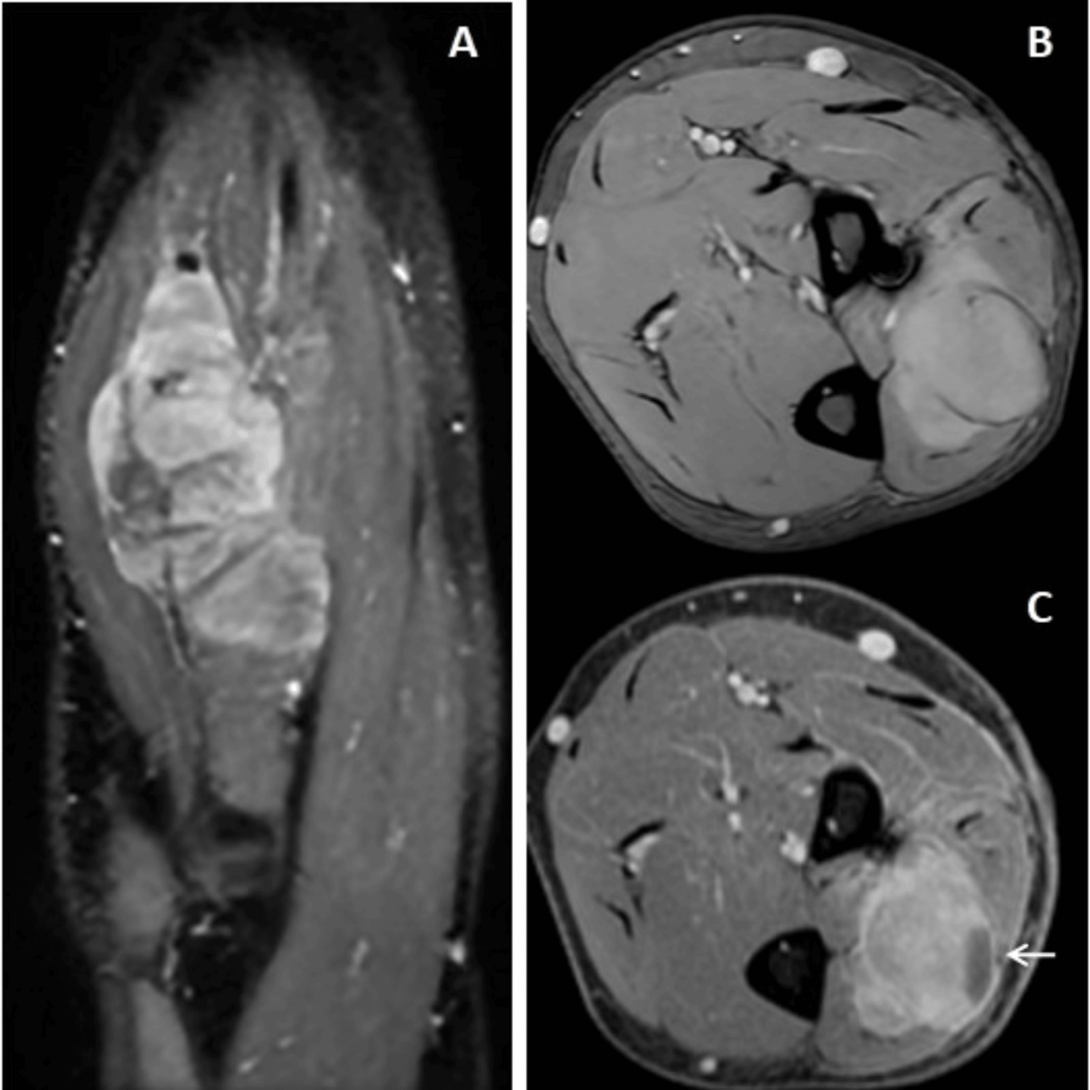
Post-contrast sagittal and axial images (A, B, and C) show a heterogeneous elongated appearing lesion with lobulations and cystic spaces.

MRI showed a well-defined altered signal intensity lesion, which is iso to mild hyperintensity heterogenous on T2-weighted MR. Post-contrast reveals moderate heterogenous enhancement with central non-enhancing areas. The picture suggests the probability of peripheral nerve sheath tumor and hemangioma, with no involvement of the nerve and muscle (Figures 1-2).

The patient has undergone fine needle aspiration cytology (FNAC) outside, showing a spindle cell with slender, elongated nuclei in a background of ill-defined eosinophilic cytoplasm.

**Figure 3:**
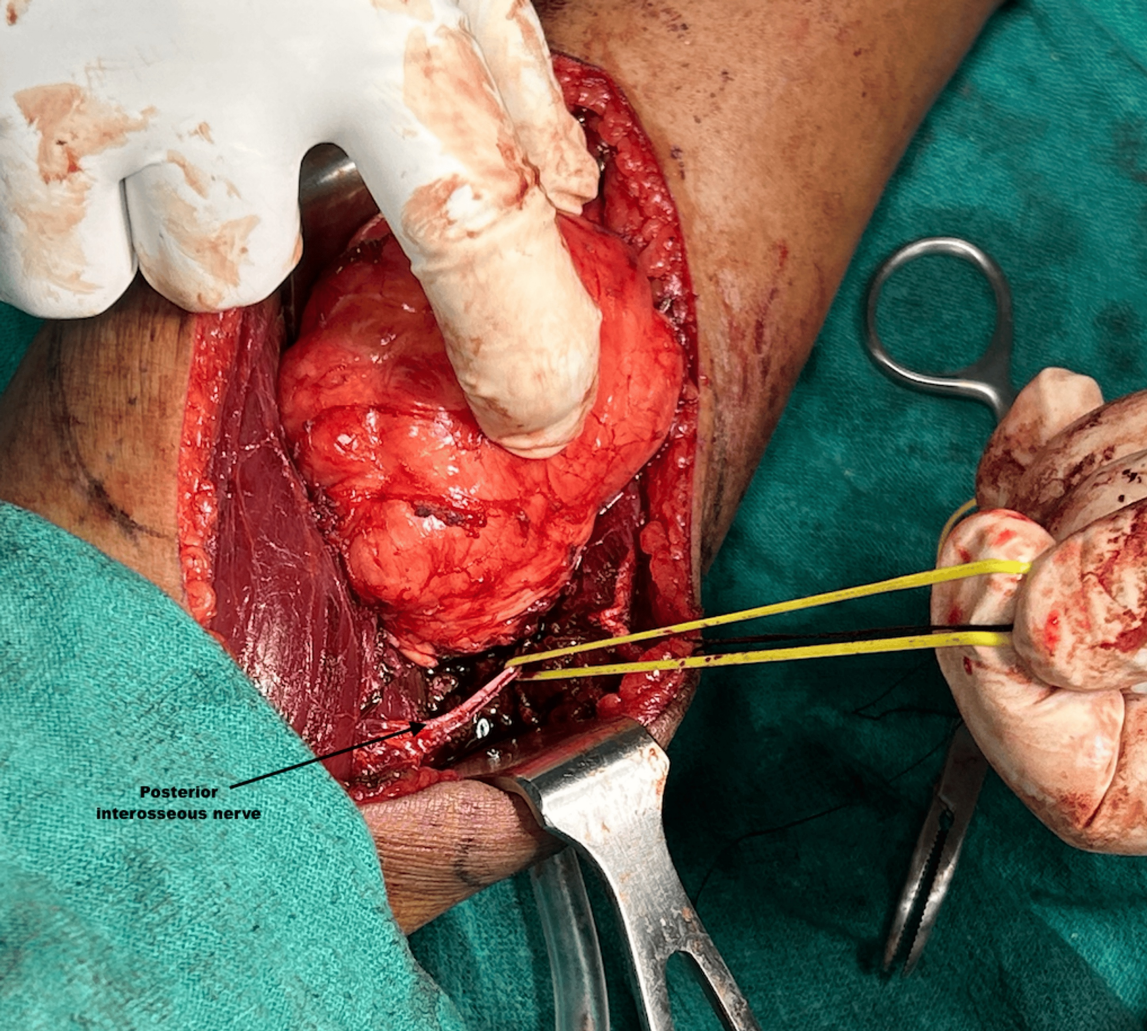
Tumor arising from posterior interosseous nerve (PIN, black arrow pointing to the PIN) and tumor retracted with a finger.

**Figure 4:**
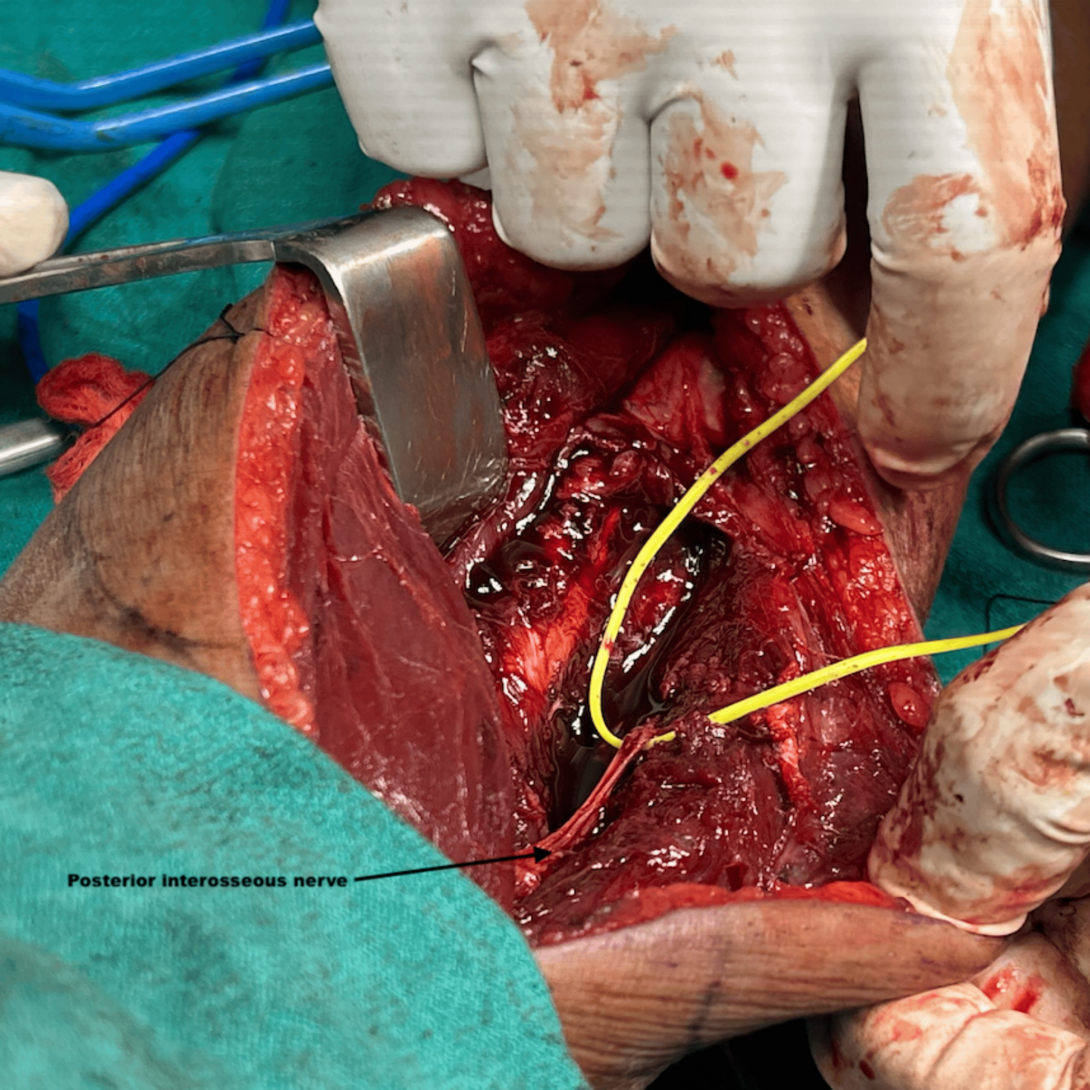
PIN after excision of the tumor.

We planned to proceed with the lesion's excision and nerve grafting if needed. The patient was counseled regarding the functional outcome and possible recurrence after the surgery. A lazy S-shaped incision was made on the right forearm, and subcutaneous tissue and superficial fascia were incised. The brachioradialis muscle was retracted, the fascia over the lesion was carefully incised, and dissection was carried out, taking care not to injure the posterior interosseous nerve (PIN). Intraoperatively, it was evident that the lesion was adherent to the posterior interosseus nerve (PIN) (Figure 3). The lesion was precisely separated from the nerve without any injury (Figure 4). The nerve course was carefully delineated, hemostasis was achieved, and the wound closed in layers. Postoperatively, weakness was observed in the right index finger. We advised a wrist drop splint over a period of one month, after which the patient regained normal movement of the finger. We followed up for a few months, and during that period, the wound was healthy without any unsightly scar and no recurrence. 

**Figure 5:**
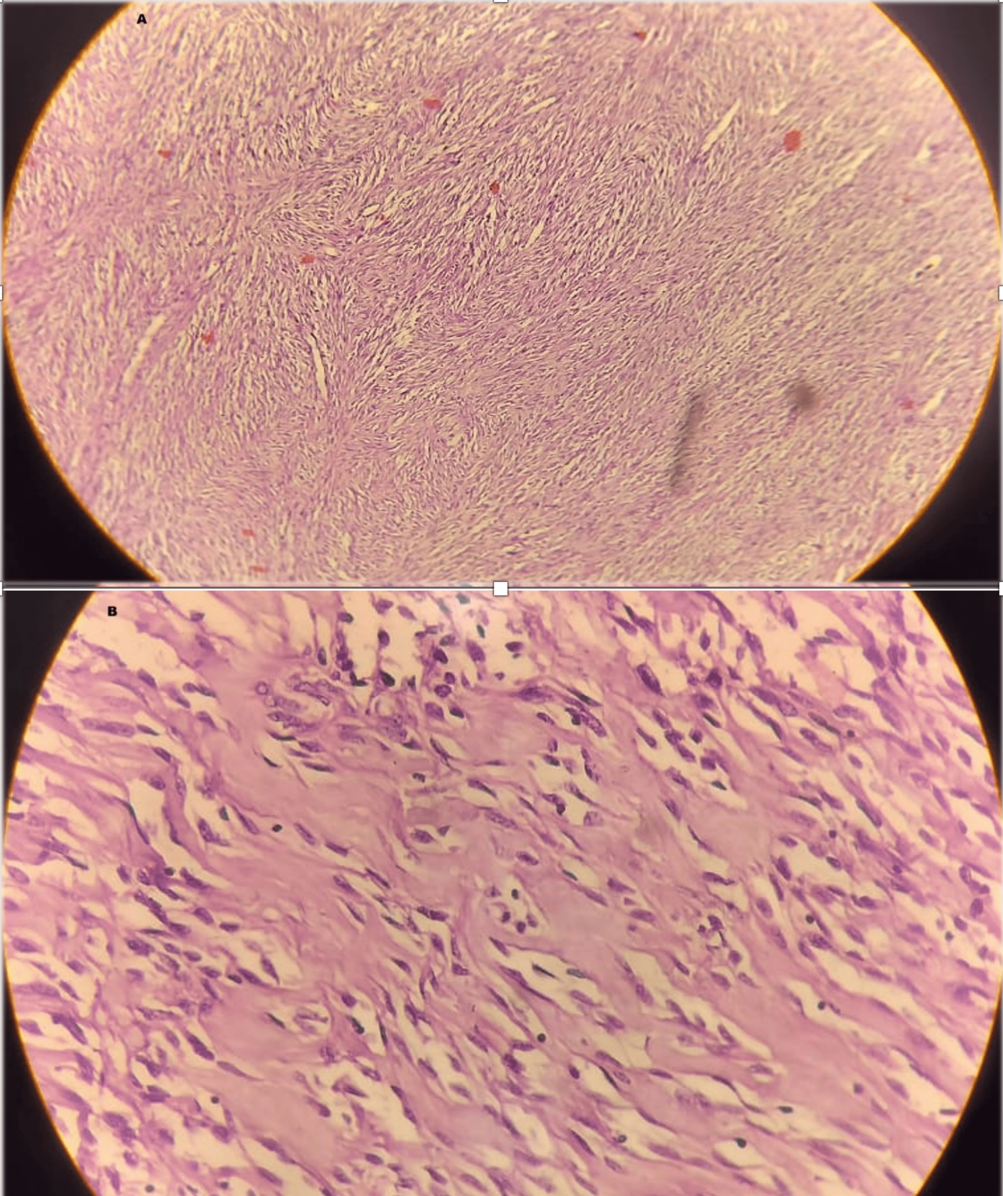
(A) A well-demarcated spindle cell tumour arranged in storiform, whorled, or short fascicular patterns (H&E 100X). (B) Individual tumor cells are slender and have a fibroblastic-like appearance with long, delicate cytoplasmic processes separated by fibrotics stroma (H&E 400x).

A biopsy shows well-demarcated spindle cell tumors arranged in storiform, whorled, or short fascicular patterns. Perineural cells are slender and have a fibroblastic-like appearance with long, delicate cytoplasmic processes (Figures 5A, 5B). Immunohistochemistry could not be performed as the patient was reluctant to go ahead with another test.

## Discussion

In varying literature, the lesion is attributed to different names, namely, localized hypertrophic mononeuropathy, interstitial hypertrophic neuropathy, pseudo-onion bulb mononeuropathy, hypertrophic neurofibroma, and intraneural neurofibroma. The multitude of nomenclature of the tumor entity is perplexing; however, historically, all those terms were taken in context to both the intraneural PN and reactive Schwann cell. It can only be differentiated by ultrastructural and immunohistochemistry studies [11].

Anatomically, the perineurium is composed of several layers arranged in a concentric pattern of flattened perineurial cells in the laminar axis around the single nerve fascicles. Intraneural PN is a rare tumor arising from the perineurial cells, and the cause is assumed to be attributed to frequent trauma. The abnormalities of chromosome 22 are also postulated to this tumor. To date, approximately the number of posterior interosseous nerves is limited to single digits (five cases), although the other site involvement is also limited and undoubtedly less reported. According to the literature, the typical age of onset of perineurium is either adolescence or young adulthood and has no sexual predilection. The symptomatic presentation is usually a painless swelling with neuropathy with progressive weakness in the affected muscles [12]. In the case report, the patient is a 38-year-old gentleman.

The sonographic picture of PN is indistinguishable from the more common benign schwannoma and neurofibroma [13]. Malignant peripheral nerve sheath tumors are distinguishable from benign entities as they infiltrate into the surrounding tissue.

MRI is nonspecific; on T1-weighted imaging with or without post-gadolinium enhancement, it shows a segmental thickening of the nerve, and T2-weighted imaging shows hyperintensity [14]. As this is a rare entity with indistinct imaging features, it is not likely that diagnostic confidence can be arrived at based on imaging alone. Regardless, initial screening with ultrasound can both provide some initial indications of the source of the problem and can effectively localize the lesion, enabling precise targeting of the lesion on MRI.

A tissue biopsy, along with an immunohistochemistry study, is the accepted standard for confirmation of the diagnosis. Typical characteristic findings of pseudo-onion bulbs, which are composed of whorls of spindle-shaped cells surrounding axon-Schwann cell complexes, interspersed among the collagen fibers. An immunohistochemistry study, usually negative for S100 and positive for epithelial membrane antigen staining and ki-67 stain, shows only a few proliferating tumor cells in the case of a perineural tumor, which may be used to further differentiate between perineural and Schwann cell origin [10].

Regarding the surgical intervention, the resection of the lesion followed by an interposition nerve graft is advisable where there is an intraoperative action potential study showing a non-functioning segment [9,10]. In our case, the conduction study could not be done; however, the nerve was carefully separated from the tumor. In approximately 5% of cases, the recurrence was noted [9].

Gluen et al. also opined that the nerve graft has to be interposed if no action potential is recorded intraoperatively [15]. Moreover, it is found that nerve recovery is quick in younger patients. Early diagnosis and identification of the lesion nerve involvement and avoiding the prolonged denervation atrophy contribute to neurological outcomes.

## Conclusions

Perineurioma is a rare entity; however, the possibility of PN should be considered in any case of swelling in the peripheral extremities with suspicion of neural origin. The prognosis is favorable in the early phase, potentially resulting in a good outcome for any neurological deficits. Though benign in nature, prognostication of recurrence has to be communicated while counseling the patient for surgery.
